# Efficient Multiple Kernel Learning Algorithms Using Low-Rank Representation

**DOI:** 10.1155/2017/3678487

**Published:** 2017-08-22

**Authors:** Wenjia Niu, Kewen Xia, Baokai Zu, Jianchuan Bai

**Affiliations:** ^1^School of Electronic and Information Engineering, Hebei University of Technology, Tianjin 300401, China; ^2^Key Lab of Big Data Computation of Hebei Province, Tianjin 300401, China

## Abstract

Unlike Support Vector Machine (SVM), Multiple Kernel Learning (MKL) allows datasets to be free to choose the useful kernels based on their distribution characteristics rather than a precise one. It has been shown in the literature that MKL holds superior recognition accuracy compared with SVM, however, at the expense of time consuming computations. This creates analytical and computational difficulties in solving MKL algorithms. To overcome this issue, we first develop a novel kernel approximation approach for MKL and then propose an efficient Low-Rank MKL (LR-MKL) algorithm by using the Low-Rank Representation (LRR). It is well-acknowledged that LRR can reduce dimension while retaining the data features under a global low-rank constraint. Furthermore, we redesign the binary-class MKL as the multiclass MKL based on pairwise strategy. Finally, the recognition effect and efficiency of LR-MKL are verified on the datasets Yale, ORL, LSVT, and Digit. Experimental results show that the proposed LR-MKL algorithm is an efficient kernel weights allocation method in MKL and boosts the performance of MKL largely.

## 1. Introduction

Support Vector Machine (SVM) is an important machine learning method [1], which trains linear learner in feature space derived by the kernel function, and utilizes generalization theory to avoid overfitting phenomenon. Recently, Multiple Kernel Learning (MKL) method has received intensive attention due to its more desirable recognition effect over the classical SVM [2, 3]. However, parameter optimization of multiple kernel introduces a high computing cost in searching the entire feature space and solving tremendous convex quadratic optimization problems. Hassan et al. [4] utilize the Genetic Algorithm (GA) to improve the search efficiency in MKL, but the availability of GA remains to be proved and its search direction is too complex to be determined. Besides, with the data volume increasing exponentially in the real world, it is intractable to solve large scale problems by using conventional optimal methods. Therefore, many approaches have been put forward to improve MKL. For example, the Sequential Minimal Optimization (SMO) algorithm [5] is a typical decomposition approach that updates one or two Lagrange multipliers at every training step to get the iterative solutions. And some online algorithms [6, 7] refine predictors through online-to-batch conversion scheme, whereas it should be noted that the convergence rate of such decomposition approaches is unstable. Another approach is to approximate the kernel matrix such as Cholesky decomposition [8, 9] which is used to reduce the computational cost, however, at the cost of giving up recognition accuracy due to lost information.

Generally, when we set *n* as sample size and *N* as the number of kernels, the complexities of solving convex quadratic optimization problems in SVM and MKL are *O*(*n*^3^) and *O*(*Nn*^3.5^) [10], respectively. It can be observed that the computing scale depends on the size of training set rather than the kernel space dimension [8]. In this big data era, it is imperative to find an approach that can minimize the computing scale while capturing the global data structure to perfect SVM or MKL. Low-Rank Representation (LRR) [11] recently has attracted great interest in many research fields, such as image processing [12, 13], computer vision [14], and data mining [15]. LRR, as a compressed sensing approach, aims to find the lowest-rank linear combination of all training samples for reconstructing test samples under a global low-rank constraint. When the training samples are sufficiently complete, the process of representing data with low-rank will augment the similarities among the intraclass samples and the differences among the interclass samples. Meanwhile, if the data is corrupted, since the rank of coefficient matrix will be largely increased, the lowest-rank criterion can enforce noise correction. LRR integrates data clustering and noise correction into a unified framework, which can greatly improve the recognition accuracy and robustness in the preprocessing stage. In this sense, the recognition of SVM or MKL can be increasingly accurate and of high speed when they are combined with LRR.

In this paper, combining LRR and MKL, we will develop a novel recognition approach so as to construct a Low-Rank MKL (LR-MKL) algorithm. In the proposed algorithm, the combined Low-Rank SVM (LR-SVM) will simultaneously be utilized as the reference. We will conduct extensive experiments on public databases to show that our proposed LR-MKL algorithm can achieve better performance than original SVM and MKL.

The remainder of the paper is organized as follows: We start by a brief review on SVM in next section. In Section 3 we describe some existing MKL algorithms and their structure frames.  Section 4 is devoted to introducing efficient MKL algorithms using LRR, which we present and call LR-MKL. Experiments, which demonstrate the utility of the suggested algorithm on real data, are presented in Section 5.  Section 6 gives the conclusions.

## 2. Overview of SVM

Given input space **X**⊆R^*D*^ and label vector **Y**, {**X**, **Y**} meets independent and identically distributed conditions, so the training set can be denoted as {**x**_*i*_, *y*_*i*_}_*i*=1_^*n*^ (contains *n* samples). According to the theory of structural risk minimization [1], SVM can find the classification hyperplane with the maximum margin in the mapping space R^*P*^. Hence, the SVM training with *l*_1_-norm soft margin is a quadratic optimization problem:

(1) min 12w,w+C∑i=1nξi,s.t. yiw,xi+b≥1−ξi, w∈RP, ξi∈R+n, i=1,…,n.

Here, **w** is the weight coefficient vector, *C* is the penalty factor, *ξ*_*i*_ is the slack variable, and *b* is the bias term of classification hyperplane. The optimization problem can be transformed into its dual form by introducing Lagrangian multiplier *α*_*i*_, *α*_*j*_, and the data **X** can be implicitly mapped to the feature space by utilizing the kernel function *K*, so formula (1) changes into

(2) min 12∑i=1n∑j=1nyiyjαiαjKxi,xj−∑i=1nαi,s.t. ∑i=1nαiyi=0, C≥αi≥0, i=1,…,n.

Simplify the objective function of formula (2) into vector form:

(3) min 12αTQα+1Tα,s.t. yTα=0, C≥α≥0,

where *α* ∈ R^*n*^ is the vector of Lagrangian multiplier, **y** ∈ **Y**^*n*^ is the label vector, 1 is a vector of 1′s (*n∗*1-dimension), and **Q**_*ij*_ = *y*_*i*_*y*_*j*_*K*(**x**_*i*_, **x**_*j*_). If the solution of optimization problem is *α*_*i*_^*∗*^, *i* = 1,…, *n*, the discriminant function can be represented as

(4) $$\begin{array}{c} f\left(\;x\;\right)=\overset{n}{\underset{i=1}{\sum }}\alpha_{i}^{*}y_{i}K\left(\;x_{i},x\;\right)+b. \end{array}$$

The kernel functions commonly used in SVM are linear kernel, polynomial kernel, radial basis function kernel, and sigmoid kernel, respectively, denoted as

(5) KLINxi,xj=xi,xj,KPOLxi,xj=γ∗xi,xj+1q,KRBFxi,xj=exp⁡−γ∗xi−xj22,KSIGxi,xj=tanh⁡γ∗xi,xj+1.

To obtain the high recognition accuracy in monokernel SVM, we need to discern what kind of kernel distribution characteristics the test data will obey. Nevertheless, it is unpractical and wasteful of resources to try different distribution characteristics one by one. In this sense, we need MKL to allocate the kernel weights based on the data structure automatically.

## 3. Multiple Kernel Learning (MKL) Algorithms

To improve the universal applicability of SVM algorithm, MKL is applied instead of one specific kernel function:

(6) $$\begin{array}{c} K_{\mu }\left(\;x_{i},x_{j}\;\right)=f_{\mu }\left(\;{\left\{\;K_{m}\left(\;x_{i},x_{j}\;\right)\;\right\}}_{m=1}^{M}\;|\;\mu \;\right), \end{array}$$

where *K*_*m*_ is the monokernel function. The multiple kernel *K*_*μ*_ can be obtained by function *f*_*μ*_ : R^*D*^ → R^*P*^ combining *M* different *K*_*m*_. And *μ* is the proportion parameter of kernel. There are many different methods to assign kernel weights.

Pavlidis et al. [16] propose a simple combination mode using an unweighted sum or product of heterogeneous kernels. The combining function of this Unweighted Multiple Kernel Learning (UMKL) method is

(7) Kμxi,xj=∑m=1MKmxi,xj,Kμxi,xj=∏m=1MKmxi,xj.

In a follow-up study, the distribution of *μ* in MKL becomes a vital limiting factor of availability. Chapelle and Rakotomamonjy [17] report that the optimization problem can be solved by a project gradient method in two alternative steps: first, solving a primal SVM with the given *μ*; second, updating *μ* through the gradient function with *α* calculated in the first step. The kernel combining function, objective function, and gradient function of this Alternative Multiple Kernel Learning (AMKL) method are

(8) Kμxi,xj∑m=1MμmKmxi,xj,Jμ12∑i=1n ∑j=1nyiyjαiαj∑m=1MμmKmxi,xj−∑i=1nαi,∂Jμ∂μm12∑i=1n ∑j=1nyiyjαiαjKμxi,xj∂μm=12∑i=1n ∑j=1nyiyjαiαjKmxi,xj∀m.

The Generalized Multiple Kernel Learning (GMKL) method [18] also employs the gradient tool to approach solution, but it regards kernel weights as a regularization item *r*(*μ*), which is taken as (1/2)(*μ* − 1/*M*)^T^(*μ* − 1/*M*). So the objective function and gradient function can be transformed into

(9) Jμ=12∑i=1n ∑j=1nyiyjαiαjKμxi,xj−∑i=1nαi−rμ,∂Jμ∂μm=12∑i=1n ∑j=1nyiyjαiαj∂Kμxi,xj∂μm−∂rμ∂μm∀m.

And the kernel combined function is

(10) Kμxi,xj∏m=1Mexp⁡−μmxim−xjm2=exp⁡∑m=1M−μmxim−xjm2.

There is another two-step alternate method using a gating model called Localized Multiple Kernel Learning (LMKL) method [19]. The formula of locally combined kernel is represented as

(11) $$\begin{array}{c} K_{\mu }\left(\;x_{i},x_{j}\;\right)=\overset{M}{\underset{m=1}{\sum }}\mu_{m}\left(\;x_{i}\;\right)\langle \;\Phi_{m}\left(\;x_{i}\;\right),\Phi_{m}\left(\;x_{j}\;\right)\;\rangle \mu_{m}\left(\;x_{j}\;\right), \end{array}$$

where Φ(**x**) is the mapping space of feature space. To ensure nonnegativity, kernels can be composed in competitive or cooperative mode by using softmax form and sigmoid form [25], respectively:

(12) softmax:  μm=exp⁡vm,x+vm0∑h=1Mexp⁡vh,x+vh0,∀m,sigmoid:  μm=1exp⁡−vm,x+vm0,∀m,

where **V** = {**v**_*m*_, *v*_*m*0_}_*m*=1_^*M*^ denotes the parameter of gating model. On the other hand, Qiu and Lane [20] quantify the fitness between kernel and accuracy in a Heuristic Multiple Kernel Learning (HMKL) way by exploiting the relationship between kernel matrix **K** and sample label **y**. The relationship can be expressed by kernel alignment:

(13) $$\begin{array}{c} F\left(\;K,yy^{T}\;\right)=\frac{{\langle K,yy^{T}\rangle }_{F}}{\sqrt{{\langle K,K\rangle }_{F}{\langle yy^{T},yy^{T}\rangle }_{F}}}=\frac{{\langle K,yy^{T}\rangle }_{F}}{n\sqrt{{\langle K,K\rangle }_{F}}}, \end{array}$$

where 〈**K**, **yy**^T^〉_*F*_ = ∑_*i*=1_^*n*^∑_*j*=1_^*n*^*K*(**x**_*i*_, **x**_*j*_)*y*_*ij*_*y*_*ij*_^T^, 〈·,·〉_*F*_ is the Frobenius inner product. Using kernel alignment weighs the proportion of multikernels:

(14) $$\begin{array}{c} \mu_{m}=\frac{F\left(K_{m},yy^{T}\right)}{\sum_{h=1}^{M}F\left(K_{h},yy^{T}\right)}\;\forall m. \end{array}$$

Then, the concentration bound is added in kernel alignment by Cortes et al. [21] to form centering kernel:

(15) $$\begin{array}{c} {\left[\;K^{c}\;\right]}_{ij}=K_{ij}-\frac{1}{n}\overset{n}{\underset{i=1}{\sum }}K_{ij}-\frac{1}{n}\overset{n}{\underset{j=1}{\sum }}K_{ij}+\frac{1}{n^{2}}\overset{n}{\underset{i,j=1}{\sum }}K_{ij}. \end{array}$$

Accordingly, the multikernel weights of this Centering Multiple Kernel Learning (CMKL) method are

(16) $$\begin{array}{c} \mu =\frac{C^{-1}a}{{\left\|C^{-1}a\right\|}_{2}}, \end{array}$$

where **C** = {〈*K*_*m*_^*c*^, *K*_*h*_^*c*^〉_*F*_}_*m*,*h*=1_^*M*^ and **a** = {〈*K*_*m*_^*c*^, **yy**^T^〉_*F*_}_*m*=1_^*M*^.

Later, Cortes et al. [22] studied a Polynomial Multiple Kernel Learning (PMKL) method, which utilized the polynomial combination of the base kernels with higher degree (*d* ≥ 1) based on the Kernel Ridge Regression (KRR) theory:

(17) Kμxi,xj=∑μk1k2⋯kMK1xi1,xj1k1·K2xi2,xj2k2⋯KMxiM,xjMkMkm≥0,  sum⁡k1+k2+⋯+kM≤d,  μk1k2⋯kM≥0.

However, the computing complex of coefficients *μ*_*k*_1_*k*_2_⋯*k*_*M*__ is *O*(*M*^*d*^), which is too large to apply in practice. So *μ*_*k*_1_*k*_2_⋯*k*_*M*__ can be simplified as a product form by nonnegative coefficients *μ*_1_^*k*_1_^*μ*_2_^*k*_2_^ ⋯ *μ*_*M*_^*k*_*M*_^, and the special case (*d* = 2) can be expressed as

(18) $$\begin{array}{c} K_{\mu }\left(\;x_{i},x_{j}\;\right)=\overset{M}{\underset{m=1}{\sum }}\;\overset{M}{\underset{h=1}{\sum }}\mu_{m}\mu_{h}K_{m}\left(\;x_{i}^{m},x_{j}^{m}\;\right)K_{h}\left(\;x_{i}^{h},x_{j}^{h}\;\right). \end{array}$$

Here, the related optimization of learning *K*_*μ*_ can be formulated as the following min-max form:

(19) $$\begin{array}{c} \underset{\mu \in \varphi }{\min}\;\underset{\alpha \in R^{n}}{\max}\;{-\alpha }^{T}\left(\;K_{\mu }+\lambda I\;\right)\alpha +2\alpha^{T}y, \end{array}$$

where *φ* is a positive, bounded, and convex set. Two bounded sets *l*_1_-norm and *l*_2_-norm are the appropriate choices to construct *φ*:

(20) φ1=μ ∣ μ≥0,  μ−μ01≤∧,φ2=μ ∣ μ≥0,  μ−μ02≤∧.

Here, *μ*_0_ and ∧ are model parameters, and *μ*_0_ is generally equal to 0 or *μ*_0_/‖*μ*_0_‖ = 1.

Other than approaches described above, inspired by the consistency between group Lasso and MKL [26], Xu et al. [23] and Kloft et al. [24] propose an MKL iterative method in a generalized *l*_*p*_-norm (*p* ≥ 1) form. They are collectively called Arbitrary Norms Multiple Kernel Learning (ANMKL) method. On the basis of duality condition ‖**w**_*m*_‖_2_^2^ = *μ*_*m*_^2^∑_*i*=1_^*n*^∑_*j*=1_^*n*^*y*_*i*_*y*_*j*_*α*_*i*_*α*_*j*_*K*_*m*_(**x**_*i*_^*m*^, **x**_*j*_^*m*^), the updated formula of kernels weight is

(21) $$\begin{array}{c} \mu_{m}=\frac{{\left\|w_{m}\right\|}_{2}^{2/\left(p+1\right)}}{{\left(\sum_{h=1}^{M}{\left\|w_{h}\right\|}_{2}^{2p/\left(p+1\right)}\right)}^{1/p}}\;\forall m. \end{array}$$

It can be seen from the formulas in this section that the operation complexity of MKL is mainly decided by **x**_*i*_. So trying to simplify the feature space is an efficient way to improve the performance of MKL. Through the optimization of basis vectors, LRR can reduce dimension while retaining the data features, which is ideal for improving MKL.

## 4. MKL Using Low-Rank Representation

### 4.1. Low-Rank Representation (LRR)

The theoretical advances on LRR enable us to use latent low-rank structure in data [27, 28]. And it simultaneously obtains the representation of all samples under a global low-rank constraint. Meantime, the LRR procedure can operate in a relatively short time with guaranteed performance.

Let the input samples space **X** be represented by a linear combination in the dictionary **A**:

(22) $$\begin{array}{c} X=AZ, \end{array}$$

where **Z** = [**z**_1_, **z**_2_,…, **z**_*n*_] is the coefficient matrix and each **z**_*i*_ is a representation coefficient vector of **x**_*i*_. When the samples are sufficient, **X** serves as the dictionary **A**. By considering the noise or tainted data in practical application, LRR aims at approximating **X** into **AZ** + **E** by the means of minimizing the rank of matrix **A** while reducing the *l*_0_-norm of **E**, in which **A** is a low-rank matrix and **E** is the associated sparse error. It can be generally formulated as

(23) minZ,E rankA+λE0,s.t. X=AZ+E.

Here, *λ* is used to balance the effect of low-rank and error term. *l*_0_-norm as NP-hard problem can be substituted for *l*_1_-norm or *l*_2,1_-norm. We choose *l*_2,1_-norm as the error term measurement here, which is defined as ${\left\|E\right\|}_{2,1}=\sum_{j=1}^{n}\sqrt{\sum_{j=1}^{n}{\left({\left[E\right]}_{ij}\right)}^{2}}$. Meantime, rank(**A**) can relax into nuclear-norm ‖·‖_*∗*_ [29]. Consequently, the convex relaxation of formula (23) is

(24) minZ,E Z∗+λE2,1,s.t. X=AZ+E.

The optimal solution **Z**^*∗*^ can be obtained via the Augmented Lagrange Multipliers (ALM) method [11].

### 4.2. Efficient SVM and MKL Using LRR

Kernel matrix remarkably impacts the computational efficiency and accuracy of SVM and MKL. How to find an appropriate variant of kernel matrix that contains both the initial label and the data geometry structure for recognition is a crucial task. Since LRR has been theoretically proved to be superior, in the sequel, we adopt LRR to transform the kernel for augmenting the similarities among the intraclass samples and the differences among the interclass samples. Moreover, a representation of all samples under a global low-rank constraint can be attained, which is more conducive to capturing the global data structure [30]. So LR-SVM and LR-MKL are two alternative techniques that we propose to use to improve the performance of SVM and MKL.

Firstly, based on the LRR theory, we improve the monokernel SVM as the reference item, from which the improvement brought by LRR can be displayed visually. The specific procedure of efficient LR-SVM is presented in Algorithm 1.

**Algorithm 1 (efficient SVM using LRR (LR-SVM)).**

  

*Input*. This includes the whole training set {**X**, **Y**}, the feature space of testing set **X**_*S*_ = [**x**_*n*+1_, **x**_*n*+2_,…, **x**_*n*+*s*_], the parameters *t*, *γ*, *C*, *q* of SVM, and the parameter *λ* of LRR.

*Step 1*. Normalize **X**, **X**_*S*_.

*Step 2*. Perform (24) procedure on the normalized **X**, **X**_*S*_ to project them on the coefficient feature space **Z**, **Z**_*S*_, respectively.

*Step 3*. Plug **Z** and the label vector **Y** into SVM for training classification model.

*Step 4*. Utilize the obtained classification model to classify the coefficient feature **Z**_*S*_ of testing set **X**_*S*_, and the discriminant function is *f*(**z**) = ∑_*i*=1_^*n*^*α*_*i*_^*∗*^*y*_*i*_*K*(**z**_*i*_, **z**) + *b*.

*Output*. Compare the actual label vector of test set **Y**_*S*_ and the prediction label vector **Y**_*P*_ to obtain the recognition results.

It is well known that SVM suffers from instability for the various data structures. Thus, MKL recognition becomes the development trend. Next, we combine LRR and MKL algorithms mentioned in the Section 3 and change binary-classification model into multiclassification model by pairwise (one-versus-one) strategy, through which a classifier between any two categories of samples (*k* is the number of categories) can be designed. Then we adopt voting method and assign sample to the category the most votes obtained. All the combined algorithms can be summarized into a frame, which is given in Algorithm 2 and we refer to it collectively as LR-MKL.

**Algorithm 2 (efficient MKL using LRR (LR-MKL)).**

  

*Input*. This includes the whole training set {**X**, **Y**}, the feature space of testing set **X**_*S*_ = [**x**_*n*+1_, **x**_*n*+2_,…, **x**_*n*+*s*_], and the parameter *λ* of LRR.

*Step 1*~*Step 2*. They are the same as the LR-SVM algorithm.

*Step 3*. Plug **Z** and the label vector **Y** into MKL to train *k*(*k* − 1)/2 classifiers with the pairwise strategy.

*Step 4*. Utilize each one of the binary MKL classifiers to classify the coefficient feature **Z**_*S*_ of testing set **X**_*S*_.

*Step 5*. According to the prediction label vectors **Y**_*P*_1__, **Y**_*P*_2__,…, **Y**_*P*_*k*(*k* − 1)/2__ vote for the category of each sample to get the multilabels **Y**_*P*_.

*Output*. Compare the actual label vector of test set **Y**_*S*_ and the prediction label vector **Y**_*P*_ to obtain the recognition results and the kernel weight vector *μ*.

## 5. Experiments and Analysis

In this section, we conduct extensive experiments to examine the efficiency of proposed LR-SVM and LR-MKL algorithms. The operating environment is based on MATLAB (R2013a) under the Intel Core i5 CPU processor, 2.53 GHz frequency parameters. The SVM toolbox used in this paper is the LIBSVM [31], which can be easily applied and is shown to be fast in large scale databases.

The simulations are performed on diverse datasets to ensure the universal recognition effect. The test datasets range over the frequently used face databases and the standard test data of UCI repository. In the simulations, all the samples are normalized first.

1. Yale face database (http://vision.ucsd.edu/content/yale-face-database): it contains 165 grayscale images of 15 individuals with different facial expression or configuration, and each image is resized to 64*∗*64 pixels with 256 grey levels.
2. ORL face database (http://www.cl.cam.ac.uk/research/dtg/attarchive/facedatabase.htm): it contains 400 images of 40 distinct subjects taken at different times, varying light, facial expressions, and details. We resize them to 64*∗*64 pixels with 256 grey levels per pixel.
3. LSVT Voice Rehabilitation dataset (http://archive.ics.uci.edu/ml/datasets/LSVT+Voice+Rehabilitation) [32]: it is composed of 126 speech signals from 14 people with 309 features, divided into two categories.
4. Multiple Features Digit dataset (http://archive.ics.uci.edu/ml/datasets/Multiple+Features): it includes 2000 digitized handwritten numerals 0–9 with 649 features.

### 5.1. Experiments on LR-SVM

In order to demonstrate the recognition performance of SVM improved by the presented LR-SVM, we carry out numerous experiments on the Yale and ORL face database. According to the different rate of training sample (20%, 30%, 40%, 50%, 60%, 70%, and 80%), we implement seven groups of experiments on each database. To ensure stable and reliable test, each group has ten different divisions randomly, and we average them as the final results. The kernel functions are *K*_LIN_, *K*_POL_, *K*_RBF_, *K*_SIG_  (*q* = 3) and *γ* = 1/*g* (*g* is the dimension of feature space).

**Figure 1:**
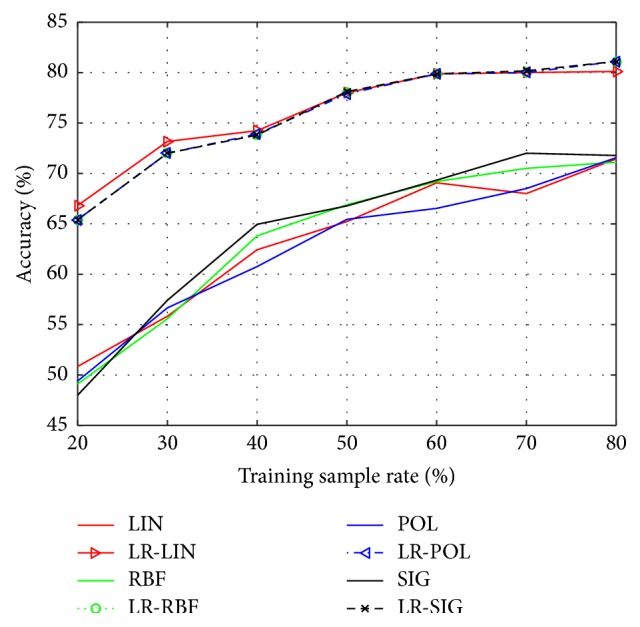
Classification accuracy of Yale by using SVM and LR-SVM.

**Figure 2:**
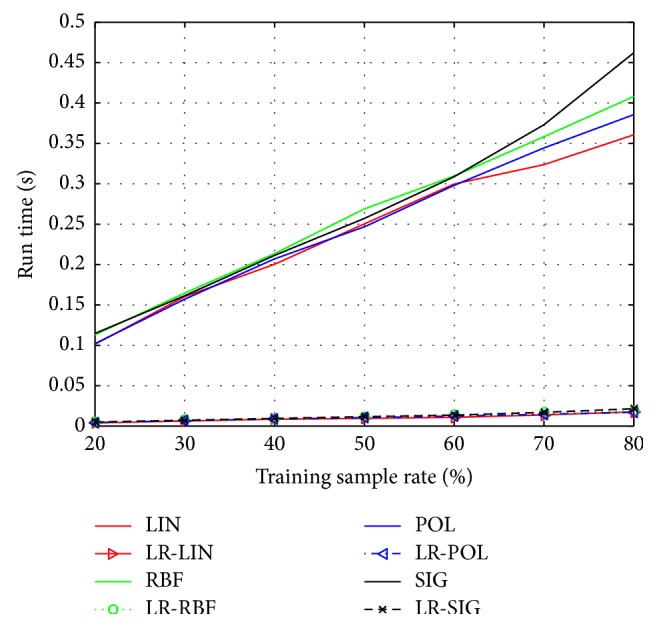
Run time of Yale by using SVM and LR-SVM.

**Figure 3:**
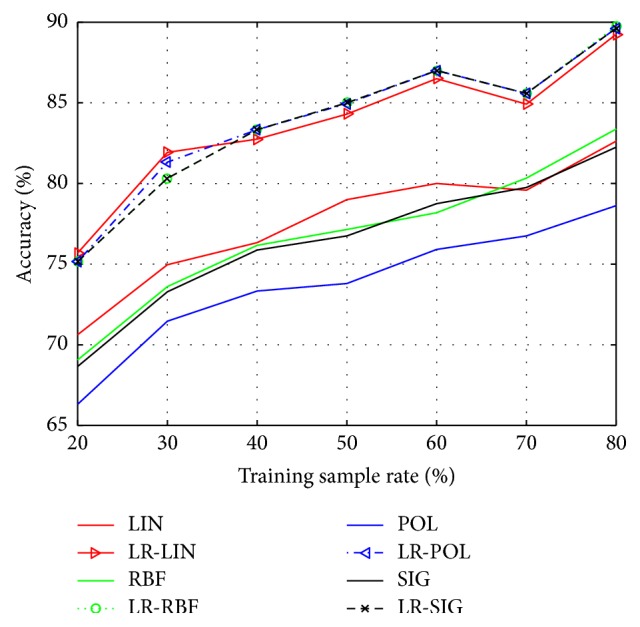
Classification accuracy of ORL by using SVM and LR-SVM.

**Figure 4:**
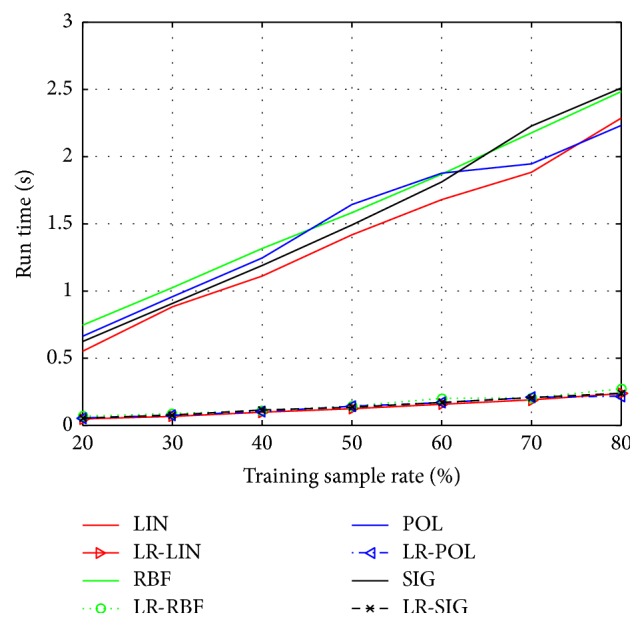
Run time of ORL by using SVM and LR-SVM.

The classification accuracy and run time of Yale database by using SVM and LR-SVM are shown in Figures 1 and 2, respectively. Similarly, the classification accuracy and run time of ORL database are shown in Figures 3 and 4. The solid lines depict the result of SVM with different kernels, while the patterned lines with the corresponding colour depict that of LR-SVM. As can be seen from the Figures 1 and 3, the proposed LR-SVM method consistently achieves an obvious improvement in classification accuracy compared to the original SVM method. In most cases, the classification accuracy increases with the rise in training sample rate. It is shown that the more complete the training set, the better the classification accuracy. But it is impossible for the training set to include so many samples in reality. LR-MKL has a high accuracy even under the low training sample rate, which is suitable for the real applications. Meanwhile, Figures 2 and 4 show that through LRR conversion, the run time can be reduced more than an order of magnitude, which is reasonable for the real-time requirements of data processing in the big data era.

### 5.2. Experiments on LR-MKL

In this section, we compare the performance of the MKL algorithms involved in Section 3 and their corresponding LR-MKL algorithms. The multikernel is composed of *K*_LIN_, *K*_POL_, *K*_RBF_, *K*_SIG_  (*q* = 3). The proportion parameter vector of kernel is *μ* = [*μ*_1_, *μ*_2_, *μ*_3_, *μ*_4_]. The comparative algorithms are listed below:

- Unweighted MKL (UMKL) [16] and LR-UMKL: (+) indicates sum form, and (*∗*) indicates product form
- Alternative MKL (AMKL) [17] and LR-AMKL
- Generalized MKL (GMKL) [18] and LR-GMKL
- Localized MKL (LMKL) [19] and LR-LMKL: (sof) distribute *μ* into softmax mode, and (sig) distribute *μ* into sigmoid mode
- Heuristic MKL (HMKL) [20] and LR-HMKL
- Centering MKL (CMKL) [21] and LR-CMKL
- Polynomial MKL (PMKL) [22] and LR-PMKL: (1) adopts the bounded set *φ*_1_ with *l*_1_-norm, and (2) adopts the bounded set *φ*_2_ with *l*_2_-norm
- Arbitrary Norm MKL (ANMKL) [23, 24] and LR-ANMKL: (1) iterates *μ* with *l*_1_-norm, and (2) iterates *μ* with *l*_2_-norm
- Besides the highest accuracy among the four monokernel SVM selected as the reference item, which is referred to as SVM(best)

We conduct experiments on the test datasets Yale, ORL, LSVT Voice Rehabilitation (LSVT, for short), and Multiple Features Digit (Digit, for short). The 60% samples of dataset are drawn out randomly to train classification model and the remaining samples serve as the test set. Through the optimized results of *γ* and penalty factor *C* by grid search method, we find that the classification accuracy varies not too much with *γ* and *C* ranging in a certain interval. So there is no need to search the whole parameter space which inevitably increases the computational cost. The penalty factor *C* can be given that trying values 0.01, 0.1, 1, 10, 100, and *γ* are fixed on 1/*g*. Then we assign a value which has the highest average accuracy on the 5*∗*2 cross validation sets to *C*. Each algorithm is conducted with 10 independent runs, and we average them as the final results. The bold numbers represent the preferable recognition effect between the original algorithms and their LRR combined algorithms. The numbers in italic font denote the algorithms whose recognition precision is inferior to the SVM(best). The recognition performance of algorithms is measured by the classification accuracy and run time, illustrated by Table 1.

In most cases, our proposed LR-MKL methods consistently achieve superior results to the original MKL, which verifies the higher classification accuracy and shorter operation time. It is indicated that LRR can augment the similarities among the intraclass samples and the differences among the interclass samples while simplifying the kernel matrix. Note that UMKL(*∗*) fails to achieve the ideal recognition effects in many cases, even less accurate than SVM(best). However, combining with LRR improves its effects to a large extent. This illustrates that simply combining kernels without according data structure is infeasible, and LRR can offset part of the consequences of irrational distribution. In general, PMKL, ANMKL, and their improved algorithms have the preferable recognition effects, especially the improved algorithms with the accuracy over 90 percent all the time. In terms of run time, it is clearly observed that the real-time performance of MKL is much worse than SVM, because MKL has a process of allocating kernel weights and the process can be very time consuming. Among them, LMKL is the worst and fails to satisfy the real-time requirement. Obviously, our combined LR-MKL can reduce the run time manifold even more than one order of magnitude, so it can speed high-precision MKL up to satisfy the real-time requirement. In brief, the proposed LR-MKL can boost the performance of MKL to a great extent.

## 6. Conclusion

The complexity of solving convex quadratic optimization problem in MKL is *O*(*Nn*^3.5^), so it is infeasible to apply in large scale problems for its large computational cost. Our effort has been made on decreasing the dimension of training set. Note that LRR just can capture the global structure of data in relatively few dimensions. Therefore, we have given a review of several existing MKL algorithms. Based on this point, we have proposed a novel combined LR-MKL, which largely improves the performance of MKL. A large number of experiments have been carried on four real world datasets to contrast the recognition effects of various kinds of MKL and LR-MKL algorithms. It has been shown that in most cases, the recognition effects of MKL algorithms are better than SVM(best), except UMKL(*∗*). And our proposed LR-MKL methods have consistently achieved the superior results to the original MKL. Among them, PMKL, ANMKL, and their improved algorithms have shown possessing the preferable recognition effects.

## Figures and Tables

**Table 1:** The performances of MKL algorithms and LR-MKL algorithms on the datasets Yale, ORL, LSVT, and Digit.

	Yale		ORL		LSVT		Digit	
	Acc	Time	Acc	Time	Acc	Time	Acc	Time
SVM(best)	69.3231	0.2981	80.0002	1.6798	76.1905	0.0142	95.9302	0.9195
LR-SVM(best)	**79.8667**	**0.0109**	**87.0015**	**0.1575**	**77.6637**	**0.0043**	**96.3304**	**0.0972**
UMKL(+) [16]	87.5114	2.5981	**93.5798**	18.2352	**78.5714**	0.0229	97.3571	4.7412
LR-UMKL(+)	**95.8936**	**0.3018**	83.4813	**1.0817**	*73.8095*	**0.0152**	**98.1595**	**2.0278**
UMKL(*∗*) [16]	*58.3248*	2.7244	*77.5022*	20.5507	*66.6935*	0.0281	96.0904	7.1661
LR-UMKL(*∗*)	**93.5739**	**0.2636**	**93.4063**	**2.1836**	***67.0033***	**0.0176**	**98.4618**	**3.6392**
AMKL [17]	85.7753	3.7236	93.8741	4.6854	80.9524	0.0452	97.4725	11.2138
LR-AMKL	**94.3799**	**0.3869**	**96.9417**	**0.4592**	**88.0952**	**0.0085**	**98.6952**	**6.8804**
GMKL [18]	86.2989	4.5330	96.2057	5.0070	85.7143	0.0565	99.1499	8.0774
LR-GMKL	**95.8015**	**0.6253**	**98.5833**	**0.7761**	**88.9286**	**0.0183**	**99.4673**	**4.5972**
LMKL(sof) [19]	87.9077	215.4055	97.0003	220.3122	85.0090	5.1989	**99.8898**	166.7978
LR-LMKL(sof)	**97.9352**	**22.7204**	**98.9724**	**17.3791**	**86.7429**	**1.1933**	98.3591	**98.1812**
LMKL(sig) [19]	88.0145	106.7552	97.0108	107.0911	88.7541	0.7238	99.3750	48.5914
LR-LMKL(sig)	**98.0667**	**15.9711**	**97.9979**	**11.7240**	**92.6627**	**0.4594**	**99.5625**	**24.5927**
HMKL [20]	*63.6037*	92.4410	93.5109	118.2340	80.5998	0.0915	97.6258	10.3559
LR-HMKL	**91.9611**	**8.5972**	**98.6893**	**10.9368**	**85.1625**	**0.0352**	**98.3479**	**6.3959**
CMKL [21]	86.4166	95.0618	96.0308	107.6940	79.9503	0.0874	96.5014	10.6074
LR-CMKL	**94.0083**	**10.6380**	**98.4799**	**12.9746**	**93.9024**	**0.0347**	**98.9113**	**6.2696**
PMKL(1) [22]	89.0035	6.1842	98.4901	6.9065	92.8571	0.1079	99.5881	24.8702
LR-PMKL(1)	**99.1429**	**0.9053**	**99.5712**	**0.9828**	**95.9386**	**0.0573**	**100.0000**	**12.9831**
PMKL(2) [22]	89.0261	5.3893	98.7533	6.5450	92.4662	0.1295	99.5046	21.1679
LR-PMKL(2)	**98.8968**	**0.8494**	**99.5828**	**0.8911**	**95.5145**	**0.0651**	**99.7941**	**13.5108**
ANMKL(1) [23, 24]	86.7210	6.4856	98.4396	20.4564	91.9827	0.1167	98.0007	10.3979
LR-ANMKL(1)	**96.8667**	**0.7041**	**99.4643**	**2.8519**	**92.2247**	**0.0247**	**99.2850**	**6.9070**
ANMKL(2) [23, 24]	86.6998	7.0664	98.2204	21.1615	**93.0035**	0.1194	98.0039	9.7753
LR-ANMKL(2)	**97.2917**	**0.8863**	**99.2857**	**3.0597**	92.5391	**0.0224**	**99.2497**	**5.0374**
